# Genetics of polymorphism in nitrogen-induced-susceptibility of rice to *Magnaporthe oryzae*

**DOI:** 10.3389/fpls.2026.1810580

**Published:** 2026-05-07

**Authors:** Yanfang Liu, Md. Hasibur Rahaman Hera, Owais Iqbal, Sauban Musa Jibril, Yi Wang, Chengyun Li

**Affiliations:** 1 State Key Laboratory for Conservation and Utilization of Bio-Resources in Yunnan, Yunnan Agricultural University, Kunming, China; 2 Quality Standards and Testing Technology Research Institute, Yunnan Academy of Agricultural Sciences, Kunming, China; 3 Yunnan-CABI Joint Laboratory for Integrated Prevention and Control of Transboundary Pests, Yunnan Agricultural University, Kunming, Yunnan, China

**Keywords:** F-box protein, GWAS, lignin, nitrogen-induced susceptibility (NIS), peroxidase, polymorphism, rice blast

## Abstract

Nitrogen fertilization is essential for crop yield but can increase susceptibility to rice blast, a phenomenon known as Nitrogen-Induced Susceptibility (NIS). The genetic architecture governing natural variation in NIS remains poorly characterized. To address this, we performed a genome-wide association study (GWAS) using a diverse panel of 268 rice accessions from the rice diversity panel 2, under controlled nitrogen regimes 0N (control), 1N (100 mg/L NH_4_NO_3_), 2N (200 mg/L NH_4_NO_3_). Our analyses defined a NIS Index (NISI), which revealed extensive phenotypic polymorphism in nitrogen responsiveness across the rice diversity panel. GWAS identified several significant loci, including a novel locus on chromosome 8 (NIS4) harboring F-box protein genes within a stress-responsive gene cluster containing eight F-box genes, a drought resistance gene, and a heat shock protein 70 gene. Physiological dissection showed that a ‘negative NIS’ phenotype (enhanced resistance under high nitrogen) correlated with a rapid, pathogen-triggered defense response, characterized by immediate induction of peroxidase activity and lignin biosynthesis. Conversely, susceptible lines exhibited delayed defense. Gene expression analysis linked active nitrogen assimilation (GS/GOGAT cycle) to susceptibility. We conclude that NIS polymorphism has a genetic component and may involve the host’s ability to redirect nitrogen resources toward early defense activation during pathogen invasion.

## 1 Introduction

Nitrogen is a key macronutrient that helps to make amino acids, proteins, and chlorophyll and so is fundamental to photosynthesis and the yield of crops. Nitrogen is a main factor driving the growth of rice crops and yields and the quality of rice grain (Ma et al., 2019; Wang et al., 2021; Dong et al., 2022; Rahmawati et al., 2026). While nitrogen fertilizers increase yield, they can also increase the susceptibility of rice crops to disease. An overabundance of nitrogen will impair the plant’s ability to defend itself from the presence of disease and thus both the severity of the disease present and the frequency of disease will be increased. This phenomenon is referred to by researchers as nitrogen-induced susceptibility (NIS) (Veresoglou et al., 2013; Fagard et al., 2014). Therefore, the system of rice production faces numerous environmental stresses that influence disease outcomes, such as climate-induced variability in rainfall and temperature (Stuecker et al., 2018). For instance, rice blast is an aggressive and damaging disease in rice, responsible for huge losses annually around the world (Iqbal et al., 2025a, b). The blast disease pathosystem associated with the rice-*Magnaporthe oryzae* interaction is a classic example of how nutrients interact with immunity, allowing scientists to use this model system to dissect the factors that can contribute to this phenomenon (Otani, 1952). High nitrogen levels can increase pathogen virulence, impair the plant’s ability to perceive salicylic and jasmonic acid defense signals, and disrupt the coordination of host defenses. As a result, disease severity in this pathosystem is enhanced (Wilson et al., 2012; Huang et al., 2017; Sun et al., 2020). Furthermore, nitrogen can also suppress partial resistance, such as that mediated by the *Pi1* gene, revealing a genetic predisposition to NIS (Ballini et al., 2013). The genetic architecture of NIS is complex and polygenic. While major resistance (*R*) genes often remain stable under high nitrogen, partial or quantitative resistance is more frequently compromised (Prabhu et al., 1996; Huang et al., 2017). Mapping efforts have identified quantitative trait loci (QTL) influencing disease outcome under high nitrogen, such as *NIS1* and *RRobN1*, confirming that NIS is a genetically modifiable trait rather than an inevitable consequence of fertilization (Ballini et al., 2013; Frontini et al., 2021). This is consistent with the observed wide natural variation in NIS responses among rice cultivars, ranging from high sensitivity to nitrogen to negligible or even resistant responses (Talukder et al., 2005; Ballini et al., 2013). Physiologically, NIS may arise from multiple mechanisms, including enhanced pathogen growth due to greater nitrogen availability in host tissues, a resource competition between host growth and defense investment, and pathogen manipulation of host nitrogen assimilation pathways (Vergne et al., 2010; Seifi et al., 2013; Fernandez and Wilson, 2012). During the early stages of an attack, key defense mechanisms, including the peroxidase-mediated oxidative burst and the lignin-driven fortification of cell walls, serve as critical determining factors (Lian et al., 2006; Yang et al., 2011; Iqbal et al., 2025a, b; Liu et al., 2026). Crucially, the presence of nitrogen can shape the progression and regulation of these pathways (Lian et al., 2006; Yang et al., 2011). The importance of early defense pathways is underscored by recent molecular studies that have identified key regulatory nodes in the rice-*M. oryzae* interaction. For instance, the rice F-box protein OsFBX156 enhances blast resistance by controlling ubiquitin-mediated degradation of defense proteins, linking protein turnover to immune activation (Zhao et al., 2024). Conversely, the pathogen counteracts host defenses through effectors such as Mobys1, which is thought to inhibit plant immunity by targeting the lignin biosynthetic enzyme OsCAD2, placing early cell wall fortification at the center of the battle (Zhao et al., 2024; Liu et al., 2024). These examples highlight the importance of early defense kinetics, which we investigate in the context of nitrogen availability. Despite progress, our understanding of NIS remains incomplete, highlighting a key challenge for ongoing research. Specifically, it remains unclear whether natural variation in NIS has a definable genetic basis that can be dissected at the genome-wide level, and how nitrogen availability modulates the timing of early defense responses across diverse genotypes. Meanwhile, bridging the gap between genetic research and agricultural extension services can enhance the adaptation of disease management strategies among farmer communities (Biswas et al., 2026). First, the genetic basis of NIS polymorphism, the broad spectrum of susceptibility responses observed across diverse germplasm remains largely unexplored at a genome-wide level. Secondly, there is not enough information regarding the timing of nitrogen’s role in activating early defense responses among different genotypes. More work needs to be done to link population-level genetic information with mechanistic physiology using an integrated approach. Based on previous observations that nitrogen can either enhance or suppress resistance, we propose a working hypothesis that NIS outcomes are determined by the timing of defense responses relative to pathogen invasion. Specifically, we hypothesize that: (i) in the pre-invasion stage, varieties that rapidly mobilize nitrogen toward cell-wall defenses (e.g., increased peroxidase activity and lignin deposition) will exhibit negative NIS (enhanced resistance under high nitrogen); whereas (ii) in the post-invasion stage, if the pathogen successfully manipulates host nitrogen metabolism before an effective defense is mounted, the outcome will be positive NIS (increased susceptibility). In order to address these gaps, we utilized a combined approach using the Rice Diversity Panel 2. Initially, we created a Nitrogen-Induced Susceptibility Index (NISI) to measure the trait in 268 accessions of rice at three nitrogen levels. Secondly, we conducted a genome-wide association study (GWAS) using high-density genotyping in order to identify the genetic locations that are associated with NIS polymorphism. Finally, we performed an analysis of the time courses of peroxidase activity and lignin biosynthesis as key physiological defense responses to NIS phenotypes in two genotypes differing in their NIS phenotype. This combined study reveals new genetic determinants of NIS, including a genetic locus with multiple F-box genes, and illustrates a time-dependent physiological process forming the nitrogen-immunity trade-off, and provides new insights for breeding rice cultivars with stable blast resistance under high nitrogen fertilization.

## 2 Materials and methods

### 2.1 Plant material and pathogen

A diversity panel of 268 rice (*Oryza sativa* L.) varieties from the Rice Diversity Panel 2 (RDP2) collection was used. The blast pathogen was *Magnaporthe oryzae* strain 95234I-1b, hereafter referred to as “491”.

### 2.2 Plant growth conditions and nitrogen treatments

Seeds from 268 varieties were surface-sterilized, germinated, and transplanted into a soil medium with a nitrogen content of 1.40% (w/w). The experimental setup consisted of three replicates of 10 seeds per variety, grown in cell trays where each cell measured 5 cm × 5 cm × 5 cm, with 105 cells per tray. These trays were maintained in 3 liters of water that was replenished daily. On the 15th day after germination, residual water was drained from the trays to standardize soil moisture levels in preparation for the subsequent procedures. All plants were grown in a controlled-environment growth chamber with a day/night temperature regime of 28°C/25°C, and relative humidity maintained at 70-80%. For inoculation, 17-day-old plants were transferred to an inoculation chamber. The relative humidity was increased to >95% for 24 hours to facilitate fungal penetration, after which it was reduced back to 70-80% for the remainder of the disease development period. The nitrogen treatments were applied on day 16 (exactly 24 hours prior to inoculation), comprising a 0N control with water only, a 1N treatment with 100 mg/L NH_4_NO_3_ (equivalent to 35 mg N/L), and a 2N treatment with 200 mg/L NH_4_NO_3_ (equivalent to 70 mg N/L). The calculation is based on the molecular weight of NH_4_NO_3_ (80 g/mol) and the mass of nitrogen within it (28 g N/mol). The soil baseline nitrogen content was 1.40% (w/w), which provided sufficient background nutrition for normal growth; the pulse treatment was designed to create a differential nitrogen environment specifically at the time of infection to assess acute effects on defense responses, minimizing confounding effects from nitrogen-induced changes in plant growth or architecture.

### 2.3 Inoculation and disease assessment

The fungal strain 491 was used to inoculate on 17-day-old plants with a spore suspension at a concentration of 10,000 spores/mL. Disease assessment was performed six days post-inoculation. To ensure consistency and minimize individual cognitive bias, a single trained evaluator assessed the disease for all 268 varieties. For this purpose, leaves from each variety were photographed to create a permanent image record. The Disease Index (DI) was calculated to quantify disease severity following the method of Hayashi and Fukuta (2009), whereby each leaf was categorized into one of five states based on lesion type and size. DI was calculated as:


DI=[∑[Number of diseased leaves in each state×State value]/[Total number of leaves × Value of the highest state]]×100

The entire experiment, from germination to disease assessment, was conducted with five biological replicates, defined as separate trays planted on different dates. Within each replicate, all three nitrogen treatments (0N, 1N, 2N) were applied independently, and each variety was represented by 10 seedlings. For physiological assays, three technical replicates per biological replicate were averaged. Outliers were identified using Grubbs’ test (α = 0.05) and removed prior to averaging. The average DI for each variety was calculated from the five biological replicates, and correlation coefficients between the average DI and nitrogen input level were analyzed using SPSS 16.0.

### 2.4 Screening for nitrogen-induced susceptibility

The 268 varieties were screened at the seedling stage based on their blast susceptibility and overall health under the experimental conditions. Varieties exhibiting robust resistance (defined as having a disease state of ‘1’ in three or more replicates across all nitrogen treatments: 0N, 1N, and 2N) were excluded. Varieties showing abnormal growth were also removed from subsequent analysis. This filtering step was deliberate to create a GWAS panel enriched for genotypes that exhibit a phenotypic shift in response to nitrogen, thereby allowing us to map the genetic basis of NIS itself. While this excludes constitutively resistant genotypes, it prevents the strong signal of major R genes from masking the more subtle genetic effects underlying NIS polymorphism.

The Nitrogen-Induced Susceptibility Index (NISI) was calculated for the remaining varieties based on the method described by Frontini et al. (2021). This index evaluates the relative effect of nitrogen on a single variety compared to its effect on the entire screened population. NISI-1 represents the assessment for the 1N treatment compared to the 0N control, while NISI-2 represents the assessment for the 2N treatment compared to the 0N control.

For a given variety ‘a’, the indices are calculated as follows:


$$\text{NISI-}1_{\text{a}}=\frac{1-\frac{{\text{DI}}_{\text{a}}^{1\text{N}}}{{\text{DI}}_{\text{a}}^{0\text{N}}}}{1-\frac{{\text{DI}}_{\text{all}}^{1\text{N}}}{{\text{DI}}_{\text{all}}^{0\text{N}}}}\text{NISI-}2_{\text{a}}=\frac{1-\frac{{\text{DI}}_{\text{a}}^{2\text{N}}}{{\text{DI}}_{\text{a}}^{0\text{N}}}}{1-\frac{{\text{DI}}_{\text{all}}^{2\text{N}}}{{\text{DI}}_{\text{all}}^{0\text{N}}}}$$

In these equations, the numerator represents the nitrogen impact on variety ‘a’, and the denominator represents the nitrogen impact on the entire screened population. Here, DIa^1N^ is the DI of variety ‘a’ under the 1N condition, and DIall^1N^ is the average DI of all screened varieties under the 1N condition. Finally, the polymorphism of NIS across the entire panel of 268 varieties was evaluated. The resulting NISI values provide a normalized measure of each variety’s response to nitrogen relative to the population: positive NISI values indicate that the variety becomes more susceptible under high nitrogen compared to the population average, while negative NISI values indicate enhanced resistance relative to the population average.

The negative denominator values for NISI-1 (- 0.077) and NISI-2 (- 0.107) mathematically reflect the population-level increase in disease severity under nitrogen (**Supplementary Table 1**). This ensures that NISI values maintain their intended interpretation: positive values indicate greater-than-average susceptibility, negative values indicate greater-than-average resistance. Filtering constitutively resistant genotypes (73 varieties with disease state ‘1’ across all nitrogen treatments) may introduce potential biases, including altered allele frequency distributions and reduced representation of resistance-associated alleles. This approach was intentionally taken to focus on the genetic basis of NIS variation rather than stable resistance, as the strong signal from major R genes could mask more subtle NIS-associated effects. To address potential concerns, we performed complementary GWAS analyses on the full panel (n = 268) using baseline resistance (DI-0N) as a covariate (**Supplementary Table 2b**). The major NIS-associated loci reported in the main text remained significant in these complementary analyses. Nevertheless, the filtered panel likely underestimates the contribution of major R genes to NIS outcomes. For validation, three alternative indices were also calculated: ΔDI (difference in DI between nitrogen treatment and control), reaction norm slope (linear regression coefficient of DI on nitrogen level), and log response ratio (ln (DI_N/DI_0N)).

### 2.5 Phenotyping and genotyping for GWAS

Genotyping data for each variety were obtained through High-Density Rice Array (HDRA) technology. Using the genotypic data (in HapMap format) and phenotypic data as input, GWAS analysis was performed with the Genomic Association and Prediction Integrated Tool (GAPIT, Version 3) (Wang and Zhang, 2021). The GAPIT pipeline was used to generate phenotypic diagnostic plots, including scatter plots, histograms, box plots, and cumulative distribution curves. For genotyping quality control, the following metrics were calculated and visualized: minor allele frequency, cumulative frequency of marker density, linkage disequilibrium (LD) decay over genomic distance, and heterozygosity frequency. The screened varieties were categorized into nine genetic subpopulations: tropical *japonica*, temperate *japonica*, admixed *japonica*, *indica*, admixed *indica*, *aus*, aromatic, admixed, and other. These varieties were collected from 46 countries and regions, which were grouped into six geographical origins: Asia, South America, North America, Africa, Europe, and Oceania. Principal component analysis (PCA) was performed on the genotypic and phenotypic data. The results were visualized in plots to illustrate the population structure according to both the nine genetic subpopulations and the six geographical types.

### 2.6 Mapping of genes associated with nitrogen-induced susceptibility

Five GWAS methods, implemented using the GAPIT package (Wang and Zhang, 2021), were applied to identify loci associated with NIS. These methods were: the General Linear Model (GLM), Mixed Linear Model (MLM), Compressed MLM (CMLM), Fixed and random model Circulating Probability Unification (FarmCPU), and Bayesian-information and Linkage-disequilibrium Iteratively Nested Keyway (BLINK). GWAS was performed using GAPIT version 3 with the following parameters: PCA = 3 (first three principal components as fixed effects), kinship matrix = centered_IBS (identity-by-state), and MAF threshold = 0.05. The FarmCPU model used the ‘CPU’ option with 3 PCs; BLINK used default settings. Significance thresholds were determined by Bonferroni correction (α = 0.05/700,000 ≈ 7.14 × 10^−8^, –log_10_(P) ≈ 7.15), with a slightly relaxed threshold of –log_10_(P) > 6 used for candidate discovery to balance stringency and power. The effectiveness of these models in accounting for population structure and familial relatedness was assessed using quantile-quantile (Q-Q) plots. In these plots, the observed negative logarithm of the *P*-values from each model is plotted against the expected distribution under the null hypothesis of no association. Furthermore, Manhattan plots were used to visualize and summarize the GWAS results, highlighting genomic regions with significant associations. The results from all five models are presented using this method. To control for constitutive resistance levels, GWAS was additionally performed on the full panel of 268 accessions (including the 73 constitutively resistant varieties) with the Disease Index under 0N (DI-0N) included as a fixed effect covariate. This analysis used the same five models and significance thresholds as described above. Due to loss of access to the original GWAS summary statistics, reference and alternative allele identities for significant SNPs could not be retrieved. However, all reported SNPs had minor allele frequencies >0.05 (**Supplementary Table 2a**), confirming these associations are not driven by rare variants.

### 2.7 Physiological assays and gene expression analysis

To investigate physiological and gene expression changes under three nitrogen regimes, four varieties representative of different NIS phenotypes were selected based on their phenotypic behavior across the nitrogen treatments (**Supplementary Table 5**): L-282 (consistently resistant, excluded from GWAS due to stable resistance across all N treatments), L-29 (negative NIS; NISI-1 = -4.2, NISI-2 = -1.0), L-157 (positive NIS; NISI-1 = 10.1, NISI-2 = 7.3), and L-82 (positive NIS with differential response between 1N and 2N; NISI-1 = 2.0, NISI-2 = 6.7). Leaf samples from these four varieties under the 0N, 1N, and 2N conditions were collected at 0, 24, 48, 72 and 96 hours post-inoculation (hpi). The samples were immediately frozen in liquid nitrogen and ground to a fine powder. From each sample, 0.1 g of powder was used to measure peroxidase (POD) activity and lignin content, following the protocols of the Micro Peroxidase Assay Kit (BC0095, Solarbio) and the Plant Lignin Enzyme-Linked Immunosorbent Assay (ELISA) Kit (Jiangsu Enzyme Industry Co., Ltd.), respectively. Additionally, one variety was selected to analyze the expression levels of key genes involved in nitrogen metabolism and defense, namely *NADH-GOGAT1* and *OsGS1–2* across the 0N, 1N, and 2N treatments.

## 3 Results

### 3.1 Disease measurement and NIS evaluation

A significant positive correlation was observed between the Disease Index (DI) and nitrogen input, with coefficients of 0.786 (1N/0N) and 0.800 (2N/0N) (p< 0.01). This confirms that nitrogen fertilization had a globally susceptible effect on the 268 varieties against infection by strain 491. Among the panel, two varieties were extremely susceptible, failing to survive inoculation. Conversely, 73 varieties consistently showed robust resistance (disease state ‘1’ in three or more replicates across all nitrogen treatments), indicating their susceptibility was not induced by nitrogen. The remaining 193 varieties, which exhibited a measurable NIS response, were selected for subsequent genetic analysis. In this subset of 193 varieties, the average DI increased by 2.3 (7.70%) under 1N and 3.2 (10.75%) under 2N, confirming the overall negative impact of nitrogen on resistance. The distribution of NIS, however, was variable. A minority of varieties showed a significant change in susceptibility (DI change > 10): 22 (11.40%) and 32 (16.58%) varieties were highly susceptible under 1N and 2N, respectively, while 10 (5.18%) and 6 (3.11%) varieties showed significantly enhanced resistance. This pattern, where strong NIS is relatively rare and the overall effect is moderate, is consistent with the findings of “Frontini et al. (2021)”. The NISI was used to quantify this effect relative to the population mean. The negative denominators for both NISI-1 (-0.077) and NISI-2 (-0.107) (**Supplementary Table 1**) mathematically reflect the global susceptibility trend. At the individual level, NISI values varied widely, from -6.429 to 20.596 (**Supplementary Table 1**), revealing a strong genotype-by-treatment interaction and affirming the panel’s suitability for GWAS. Such extreme values likely reflect combinations of factors including differential nitrogen uptake efficiencies, variation in basal defense capacity, and potentially the presence or absence of specific NIS-associated alleles. The negative extreme (enhanced resistance under nitrogen) may represent genotypes with particularly effective nitrogen-mediated defense priming, while the positive extreme (dramatically increased susceptibility) could indicate genotypes where nitrogen strongly favors pathogen growth or suppresses immunity. Varieties were categorized based on their NISI (**Supplementary Table 3**). For NISI-1, 20.7% of varieties had values >3 (positive NIS) and 9.3% had values< -3 (negative NIS). For NISI-2, 26.4% had values >3 and 2.1% had values< -3. Approximately 30% of varieties had NISI values near zero (-1 to 1) for both indices, indicating their response mirrored the global average. Representative extremes included ‘KHARSU 80::IRGC28016-1’ (high NISI-1 and NISI-2), ‘KYEEMA::GERVEX 1656-C1’ (very high NISI-1), and ‘NCS 183::IRGC51923-1’ (negative NISI, indicating nitrogen-enhanced resistance).

To validate whether NISI captures nitrogen-dependent changes in disease severity in a manner consistent with established metrics, three alternative indices were also calculated: ΔDI (difference in Disease Index between nitrogen treatment and control), reaction norm slope (linear regression coefficient of Disease Index on nitrogen level), and log response ratio (ln (DI_N/DI_0N)). NISI-1 showed strong positive correlations with ΔDI (r = 0.94, P< 0.001), reaction norm slope (r = 0.89, P< 0.001), and log response ratio (r = 0.91, P< 0.001). Similarly high correlations were observed for NISI-2 (r = 0.93, 0.90, and 0.92, respectively; **Supplementary Table 7**). Shapiro-Wilk tests showed moderate deviation from normality for NISI-1 (W = 0.962, P = 2.1 × 10^−5^) and NISI-2 (W = 0.971, P = 3.4 × 10^− 4^), consistent with the presence of extreme phenotypes (**Supplementary Table 3**). Bootstrap resampling (1,000 iterations) yielded narrow 95% confidence intervals (NISI-1: –0.21 to 0.18; NISI-2: –0.15 to 0.12), indicating robustness to sampling variation. Removal of outliers (NISI > 3 SD from mean; 8 for NISI-1, 5 for NISI-2) did not alter the major GWAS associations (**Supplementary Table 2a**).

### 3.2 Phenotyping and genotyping diagnosis

**Figure 1:**
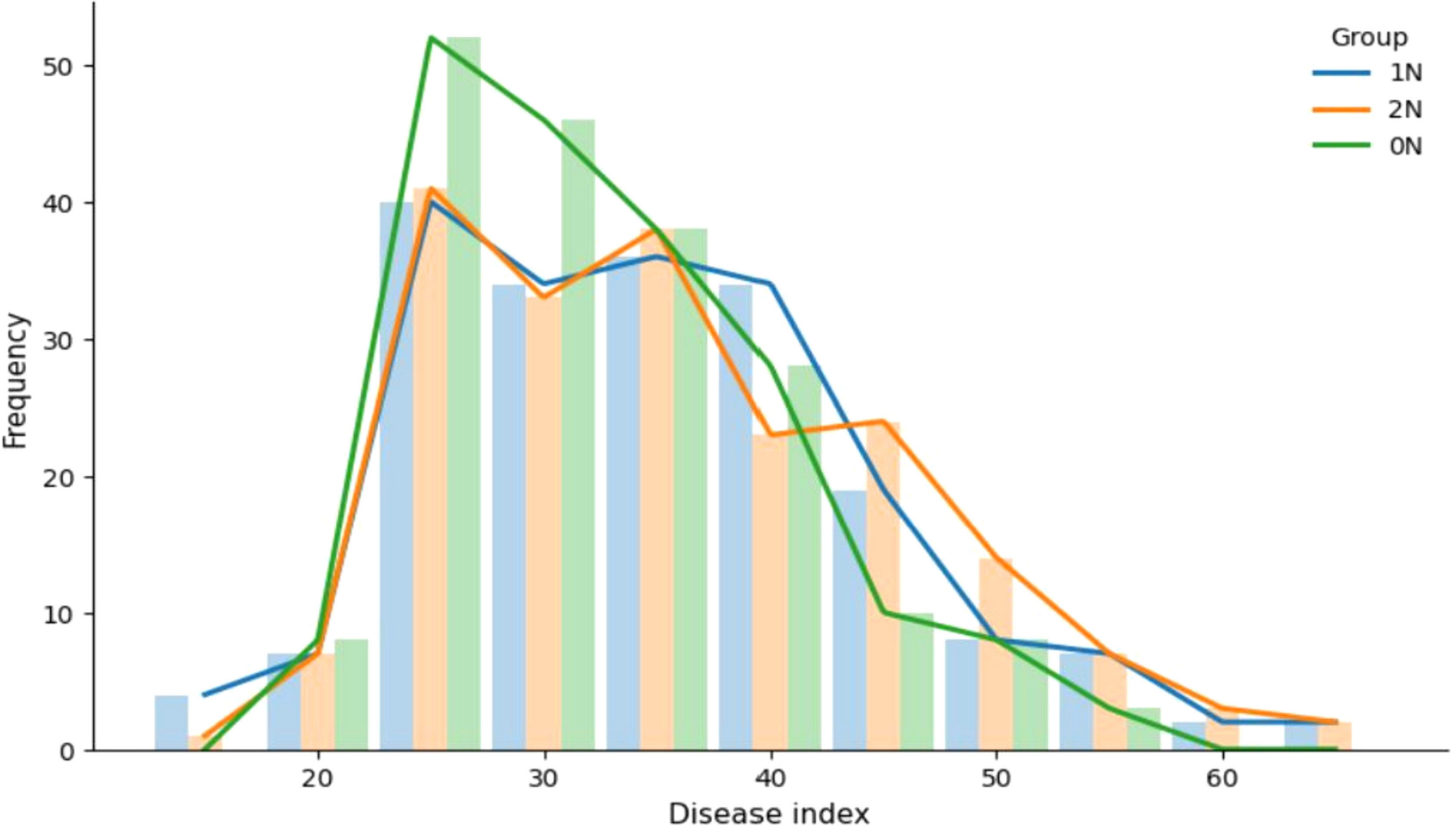
Quantity of varieties with different disease indexes (DIs) under 0N, 1N and 2N conditions. DIs of the 193 varieties were investigated after infection of rice blast 95234I-1b responding to 0N, 1N and 2N regimes. Y-axis represents variety quantity and X-axis indicates DIs.

Phenotypic analysis revealed a clear global trend toward increased susceptibility with higher nitrogen. **Figure 1** reveals as nitrogen levels rose (from 0N to 1N to 2N), the distribution of varieties consistently showed higher DI values. This was particularly evident under the 2N condition, where a distinct peak emerged in the DI 45–50 range. Furthermore, the distribution extended to more severe disease classes (DI 60-65) under 1N and 2N, with a greater number of varieties reaching DI 60 under 2N. Notably, an increase in variety frequency was also observed at a low DI of 15 under 1N and 2N treatments. This suggests that for a subset of highly resistant varieties, nitrogen application may have further enhanced their resistance, shifting them from a phenotype with several lesions (e.g., DI ~20%) to one with even fewer (DI< 20%). This pattern of nitrogen-enhanced resistance corresponds to the ‘negative NIS’ phenotype we define later (NISI< -3), indicating that approximately 2-9% of varieties in our panel exhibit this beneficial response to nitrogen (see Section 3.1 and **Supplementary Table 3**). This enhanced resistance may result from a combination of major resistance genes and a high level of partial resistance (Ballini et al., 2013).

**Figure 2:**
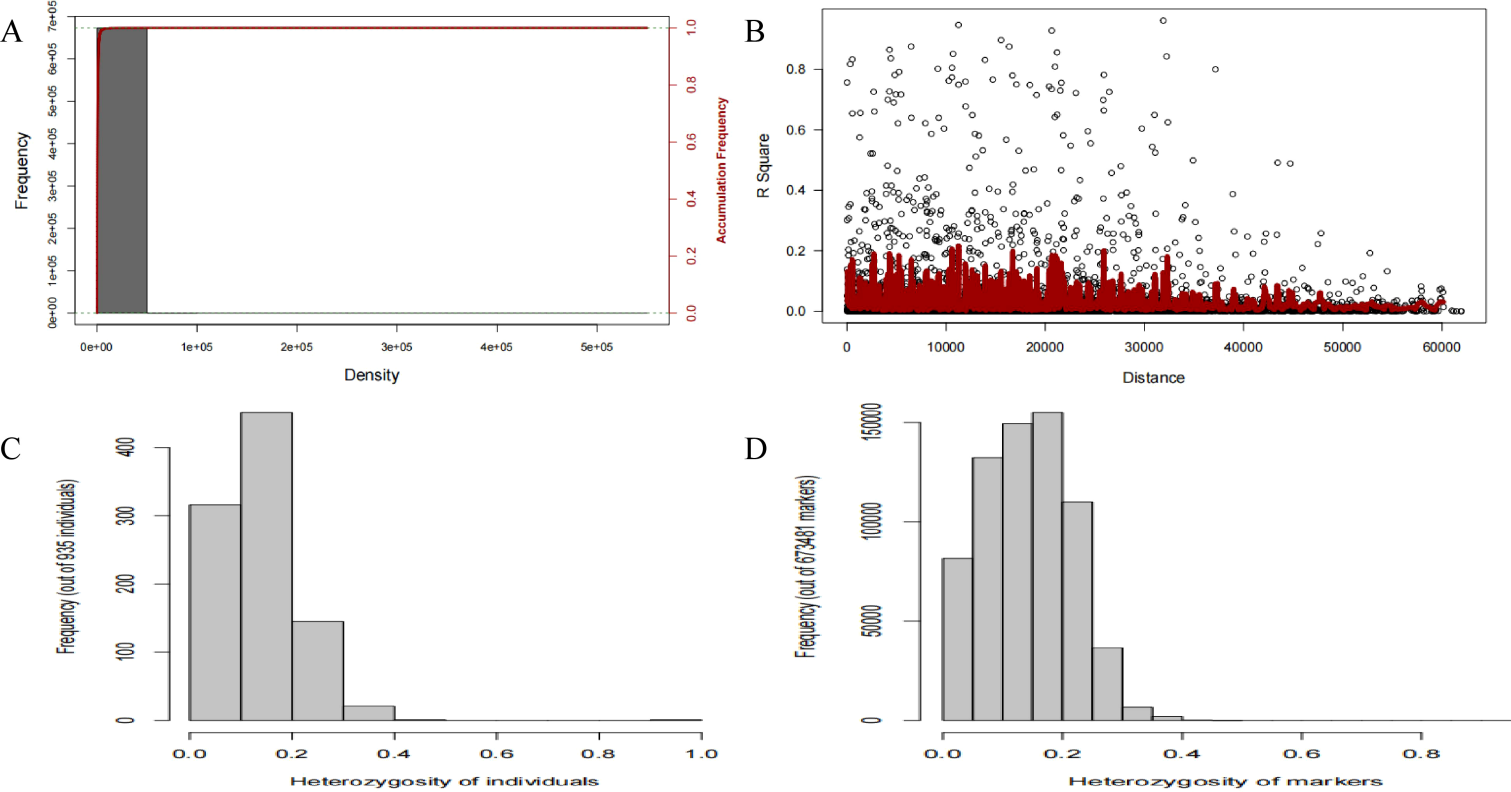
Genotype diagnosis of the HDRA data of the 193 rice varieties. **(A)** frequency and accumulative frequency of marker density. **(B)** linkage disequilibrium (LD) decay over distance. LDs were calculated as r^2^ for pair wise markers and plotted against their distance. The average LD decay, defined as the distance at which the smoothed r^2^ curve drops to half its maximum value, was approximately 200 kb, which is consistent with previous studies in diverse rice panels and provides a high mapping resolution. **(C, D)** heterozygosity of individuals/markers.

Genotyping diagnosis confirmed the high quality of the data for GWAS. The high density of HDRA markers resulted in excellent genome coverage, as demonstrated by the linkage disequilibrium (LD) decay pattern (**Figures 2A, B**). Genomic Association and Prediction Integrated Tool (GAPIT) was employed (Wang and Zhang, 2021). Additionally, the low rates of heterozygosity for both individuals and markers (below 50%, **Figures 2C, D**) confirmed the high-quality genotyping data. Therefore, both the phenotypic data from the screened varieties and the corresponding HDRA genotyping data are robust and suitable for mapping NIS-associated genes.

**Figure 3:**
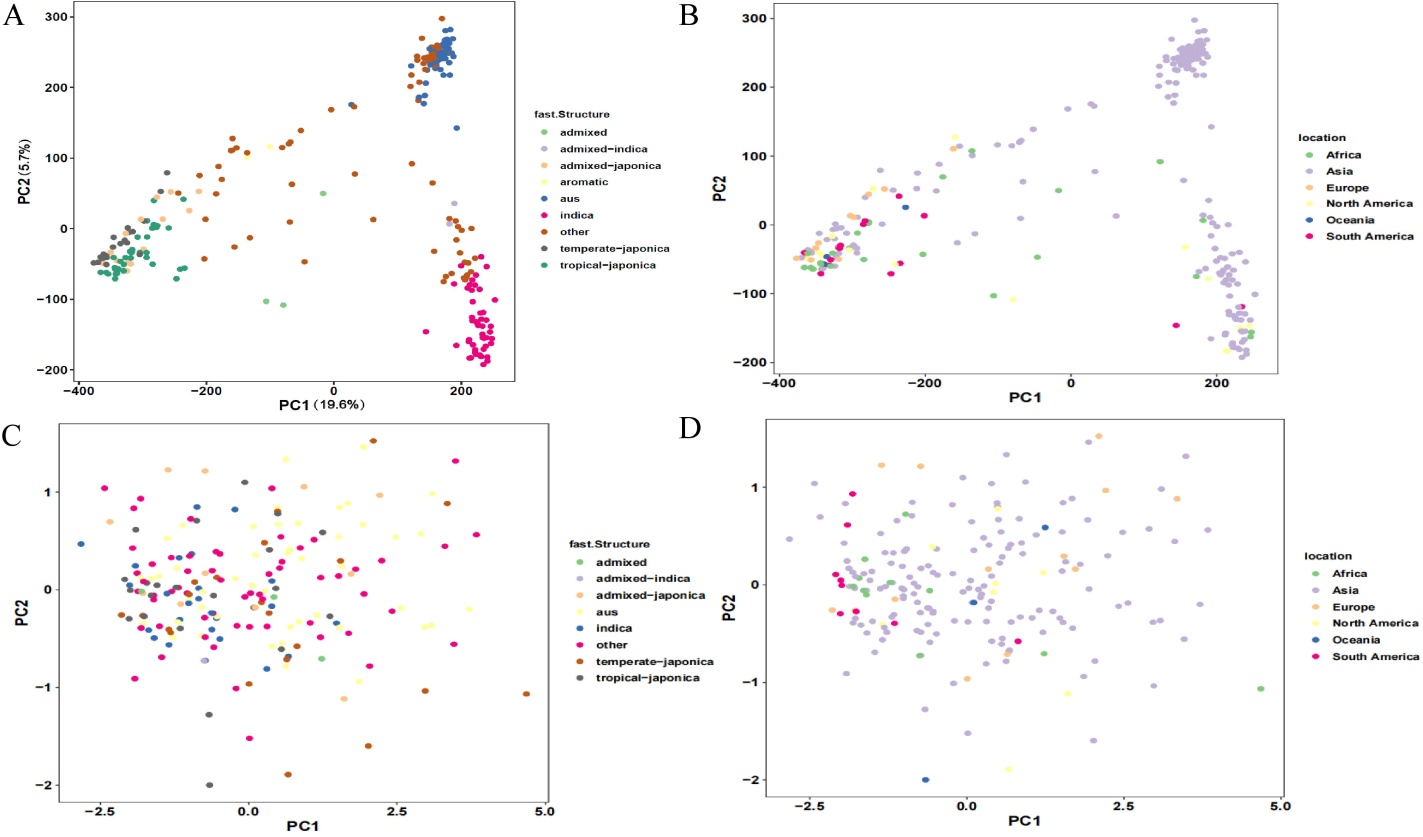
Genetic structure and phenotypic structure of the 193 rice varieties. HDRA genotyping data and NISI-2 phenotyping data of the 193 varieties were employed for principal component analysis. **(A)** PCA showing global genetic variation of the 193 varieties regarding the 9 sub-groups. **(B)** genetic variation regarding the 6 geographical distributions. **(C)** phenotypic variation regarding the 9 sub-groups. **(D)** phenotypic variation regarding the 6 geographical distributions.

The analysis of the genetic data confirmed the known major divisions between the indica, japonica, and aus rice types, as expected (**Figures 3A, B**). The more interesting finding, however, came when we looked at the NIS trait. Unlike the clear genetic groups, the plants showing the NIS phenotype (NISI-2) didn’t cluster by their ancestry or where they were grown (**Figures 3C, D**). This tells us that susceptibility to nitrogen isn’t simply tied to a plant’s broad genetic family or its geography. Indeed, the principal components capturing genetic variation explained minimal phenotypic variance (PC1 and PC2 collectively accounted for< 5% of NISI-2 variation), indicating that the genetic basis for this trait is more complex and is scattered across the diverse panel of rice we studied.

### 3.3 Mapping of genes associated with NIS

**Figure 4:**
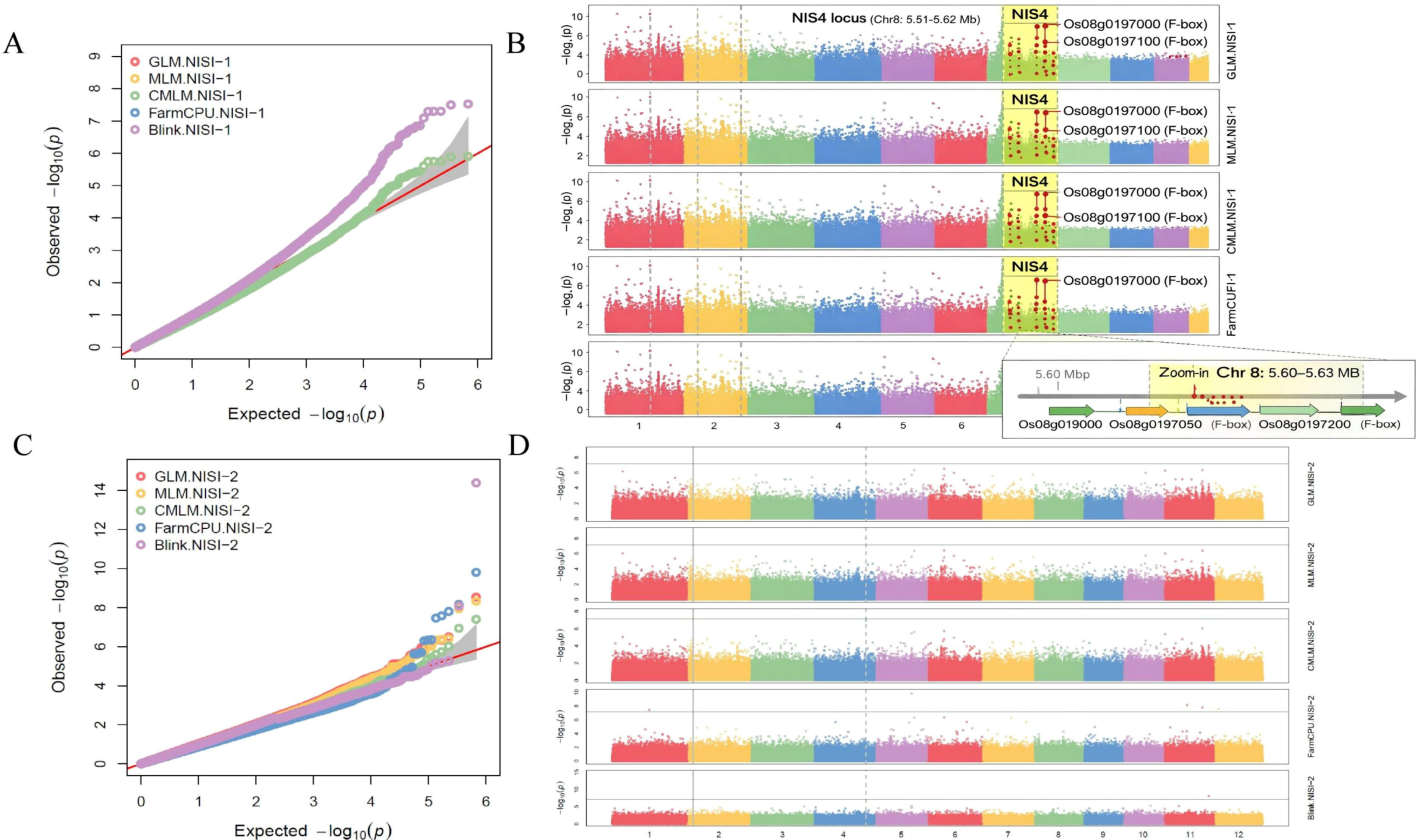
QQ plots of *P*-values and Manhattan plot from genome-wide association mapping of NIS loci. **(A, C)** quantile-quantile plot of *P*-values of NISI-1 and NISI-2, respectively. Y-axis is the observed negative base logarithm of the *P*-values, and X-axis is the expected observed negative base of 10 logarithm of the *P*-values under the assumption that *P*-values follow a uniform distribution. **(B, D)** Manhattan plot from genome-wide association mapping of NISI-1 loci and NISI-2 loci, respectively. X-axis is the genomic position of each SNP, and Y-axis is the negative logarithm of the *P*-value obtained from the GWAS model. The NIS4 locus on chromosome 8 (5.61–5.62 Mb) is highlighted by a shaded region. Red points represent highly significant SNPs within the locus (PVE: 9.8–13.2%). Vertical arrows indicate candidate genes *Os08g0197000* and *Os08g0197100* encoding F-box domain-containing proteins. The association signal is consistently detected across all five GWAS models. All significant SNPs had minor allele frequencies (MAF) > 0.05, ranging from 0.08 to 0.42, with the majority in the 0.10–0.35 range, indicating the associations are not driven by rare variants that might produce spurious signals.

To identify genetic loci associated with NIS, we performed a GWAS using five models in GAPIT. Quantile-quantile (Q-Q) plots for NISI-2 showed that the models, particularly CMLM and BLINK, effectively controlled for population structure, as evidenced by the close alignment of most data points to the diagonal (**Figures 4A, C**). The significant deviations at high -log10(p) values represent SNPs associated with the NIS trait. A genome-wide significance threshold of -log_10_(P) > 6 was applied. This stringent threshold was derived from a Bonferroni correction for multiple testing, which for our HDRA SNP dataset (approximately 700,000 markers) corresponds to a corrected α of 0.05/700,000 ≈ 7.14 × 10^−8^, or -log_10_(P) ≈ 7.15. Our threshold of 6 is slightly less stringent than this, providing a balanced approach to identifying robust associations while minimizing false positives. We also note that SNPs identified by multiple GWAS models (e.g., the seven SNPs at the NIS4 locus) provide additional confidence in these associations. Manhattan plots revealed several genomic regions with strong associations for NISI-1 and NISI-2 (**Figures 4B, D**, respectively). Using a significance threshold of -log10(p) > 6, we identified 66 and 42 significant SNPs for NISI-1 and NISI-2 (**Supplementary Table 2a**) (**Supplementary Table 2b** confirms these loci in the full-panel analysis with DI-0N covariate). To assess the influence of genotype filtering, complementary GWAS on the full panel of 268 accessions with DI-0N as a covariate. The key loci described below remained significant in this analysis (**Supplementary Table 2b**), confirming their association with NIS independent of constitutive resistance. The phenotypic variation explained (PVE) by these SNPs ranged from 8.5% to 20.1%. Key loci are summarized in **Supplementary Table 4**, and the complete list of PVEs for all significant SNPs is provided in **Supplementary Table 2a**. Among these, SNP-2.3073421 on chromosome 2 had the largest phenotypic variation explained (PVE) of 20.06% for NISI-2. This SNP lies within a region (2.94 – 3.14 Mbp) containing 19 genes that encode leucine-rich repeat receptor-like kinases (LRR-RLKs), which are central regulators of plant immune responses.

On chromosome 8, a significant candidate QTL for NISI-1 was discovered and named NIS4. This locus spans a 13-kilobase region (5.61–5.62 Mbp) and contains 10 highly significant SNPs, with phenotypic variation explained (PVE) ranging from 9.8% to 13.2%. Seven of these SNPs were significant in all five GWAS models, indicating a robust association. The NIS4 locus comprises four genes. Three of them *Os08g0197000*, *Os08g0197050*, and *Os08g0197100*, have previously been associated with seedling blast resistance. Notably, *Os08g0197000* and *Os08g0197100* encode F-box domain-containing proteins. In rice, F-box proteins such as OsFBX156 are known regulators of ubiquitin-mediated degradation of defense-related proteins and hormone receptors, influencing ROS production and PR gene expression (Zhao et al., 2024). Public expression data (RiceXPro) show that *Os08g0197000* and *Os08g0197100* are induced 2.5- to 4-fold at 24–48 hours after *M. oryzae* inoculation, supporting their candidacy as regulators that may integrate nitrogen status with immune signaling.

While direct evidence linking these F-box proteins to nitrogen-modulated blast resistance is lacking, the nutritional control of immunity suggests that NIS4 may represent a putative signaling hub that could combine nutrient sensing with immune response based on its gene content and genomic context. F-box proteins are components of SCF ubiquitin E3 ligase complexes and participate in both abiotic and biotic stress responses, including the regulation of pathogenesis-related genes and stomatal closure. The broader region around NIS4 (5.55–5.65 Mbp) is enriched for stress-related genes, including eight F-box genes, a drought resistance gene (*Os08g0196700*), and a heat shock protein 70 gene (*Os08g0197700*). The NISI-2 contained two important SNPs within the gene *Os06g0275500*. Polycomb repressive complex 2 (PRC2), a complex of proteins that controls gene activity, has a key part which is this gene. The two nearby genes encode a peroxidase precursor and a zinc finger protein, an amino acid transporter and a protein containing an FHA domain. The major locus of NISI-1 on the long arm of chromosome 10 (16.83-16.87 Mbp) is situated near the previously recorded NIS3 locus and near the SNP-10.15352978, the major NISI-2 SNP, at 15.42 Mbp, as demonstrated in **Supplementary Table 2a**. The GWAS data indicate that the genetic bases of NISI-1 and NISI-2 are not the same with the different genetic loci associated with each index (**Figures 4B, D**). To assess whether the identified NIS-associated loci were influenced by the removal of constitutively resistant genotypes, we performed a complementary GWAS on the full panel of 268 accessions, including DI-0N as a covariate to control for baseline resistance. All major loci, including NIS4 on chromosome 8 and the LRR-RLK cluster on chromosome 2, remained significant (–log_10_(P) > 6; **Supplementary Table 2b**), confirming the robustness of these associations.

Using the genotyping data from our 193-variety panel (**Supplementary Table 2a**), the genotypes for the seven highly significant SNPs within the NIS4 region (chr8: 5,610,000–5,630,000 bp) were extracted. Based on the combination of alleles at these SNPs, three major haplotypes were identified:

- Haplotype A (n = 67): associated with positive NIS (mean NISI-1 = 5.2 ± 1.8)
- Haplotype B (n = 89): associated with intermediate NIS (mean NISI-1 = 0.8 ± 0.6)
- Haplotype C (n = 37): associated with negative NIS (mean NISI-1 = –2.1 ± 0.9)

ANOVA showed significant differences among haplotypes (F = 12.4, df = 2, 190, P< 0.001), and pairwise Tukey HSD tests confirmed that all three haplotypes were significantly different from each other (P< 0.01 for all comparisons). These results provide indirect evidence that natural variation at the NIS4 locus is associated with differential NIS responses. The full results are presented in **Supplementary Table 6**.

### 3.4 Nitrogen-induced physiological and gene expression changes in selected varieties

To understand the physiological basis of NIS, we examined two key defense responses, peroxidase (POD) activity and lignin deposition in four rice varieties that represent the range of NIS phenotypes (**Supplementary Table 5**). Our selections included two varieties (L-157 and L-82) that show a positive NIS, meaning they become more susceptible under high nitrogen. For contrast, we included L-29, which exhibits a negative NIS and becomes more resistant with added nitrogen, alongside a consistently resistant variety, L-282, as a stable benchmark. A clear negative correlation was observed between overall defense marker levels and NIS. The resistant variety L-282 consistently showed the highest POD activity and lignin content, followed by L-29, L-157, and finally the susceptible L-82.

### 3.5 POD activity correlates with NIS phenotype

**Figure 5:**
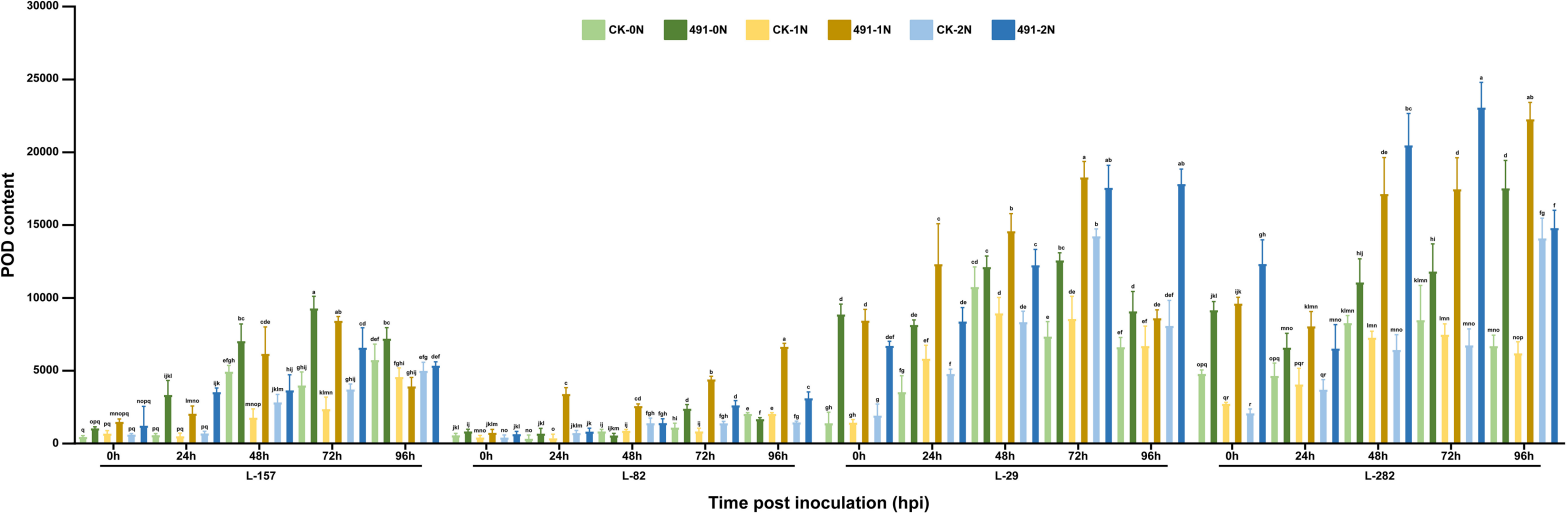
Expression patterns of peroxidase (POD) of the 4 varieties under 3 nitrogen conditions (0N, 1N and 2N) after 0h, 24h, 48h, 72h and 96h post inoculation of rice blast 95234I-1b. Varieties represent different NIS phenotypes: L-282 (resistant benchmark), L-29 (negative NIS), L-157 (positive NIS), and L-82 (positive NIS, high NISI-2). Error bars represent SD ± of the n = 5 biological replicates, and different letters above the bars showed statistical differences (P<0.05).

POD activity was induced by *M. oryzae* infection in all varieties, with peaks at 48 and 72 hours post-inoculation (hpi) (**Figure 5**). Critically, the response dynamics and magnitude were associated with NIS status. The negative-NIS varieties examined (L-282 and L-29) mounted an earlier and stronger POD response at 0–24 hpi compared to the susceptible varieties tested (L-157 and L-82). Furthermore, in these resistant varieties, nitrogen application (1N, 2N) strongly potentiated the pathogen-induced POD burst compared to the 0N condition. In contrast, the susceptible variety L-157 showed higher POD induction under 0N than under nitrogen-amended conditions. L-82 exhibited a unique pattern, with a notable POD increase under 1N but a failed response under 2N, correlating with its high NISI-2 value. Statistical analysis using two-way repeated measures ANOVA revealed a significant variety × nitrogen × time interaction for POD activity (F = 8.34, df = 24,120, P< 0.001). *Post-hoc* Tukey’s HSD tests showed that the negative-NIS variety L-29 had significantly higher POD activity at 24 hpi under 1N and 2N compared to the positive-NIS varieties L-157 and L-82 (P< 0.01).

### 3.6 Lignin deposition is enhanced by nitrogen in resistant varieties

**Figure 6:**
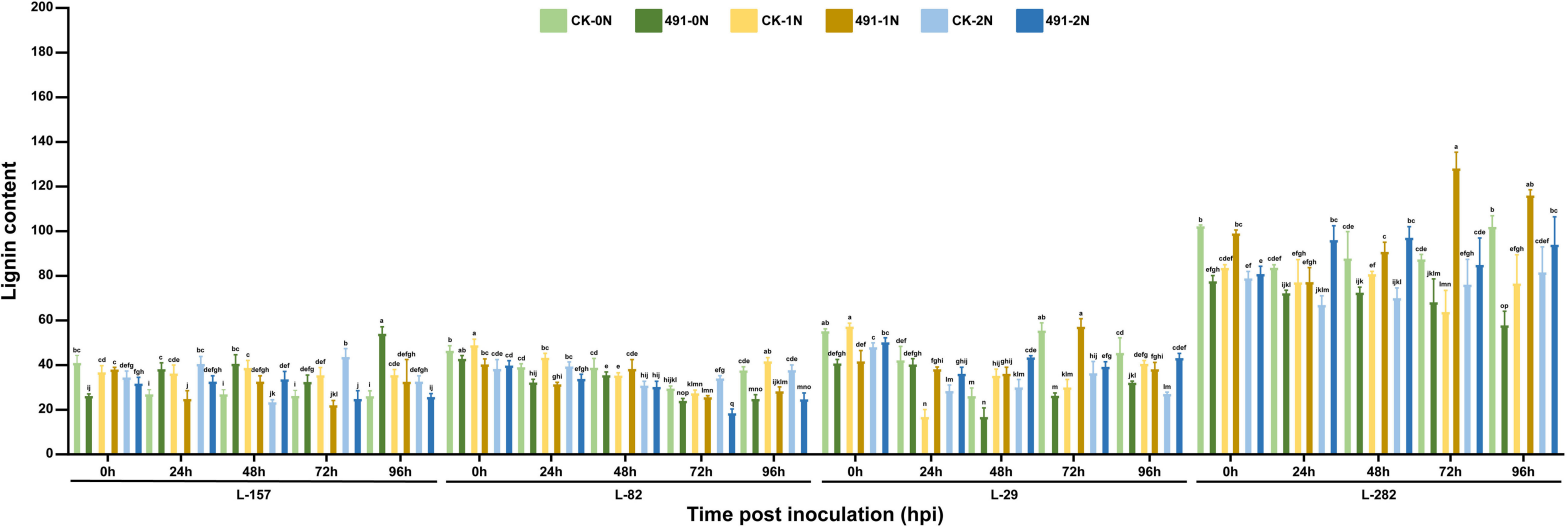
Expression patterns of lignin of the 4 varieties under 3 nitrogen conditions (0N, 1N and 2N) after 0h, 24h, 48h, 72h and 96h post inoculation of rice blast 95234I-1b. Varieties represent different NIS phenotypes: L-282 (resistant benchmark), L-29 (negative NIS), L-157 (positive NIS), and L-82 (positive NIS, high NISI-2). Error bars represent SD ± of the n = 5 biological replicates, and different letters above the bars showed statistical differences (P<0.05).

Lignin deposition showed a similar negative correlation with NIS (**Figure 6**). In the resistant varieties L-282 and L-29, nitrogen application enhanced pathogen-induced lignin biosynthesis, as lignin levels in inoculated leaves (491-) under 1N and 2N conditions were significantly higher than in both their respective controls (CK-) and the 0N treatment. This explains their fewer lesions and lower DI under high nitrogen. Conversely, in the susceptible varieties L-157 and L-82, lignin levels in control leaves were often higher than in inoculated leaves under nitrogen treatments, indicating that additional nitrogen impaired their ability to fortify cell walls upon pathogen challenge. Two-way repeated measures ANOVA for lignin content similarly showed a significant variety × nitrogen × time interaction (F = 6.21, df = 24,120, P< 0.001).

### 3.7 Nitrogen metabolism gene expression in a susceptible variety

**Figure 7:**
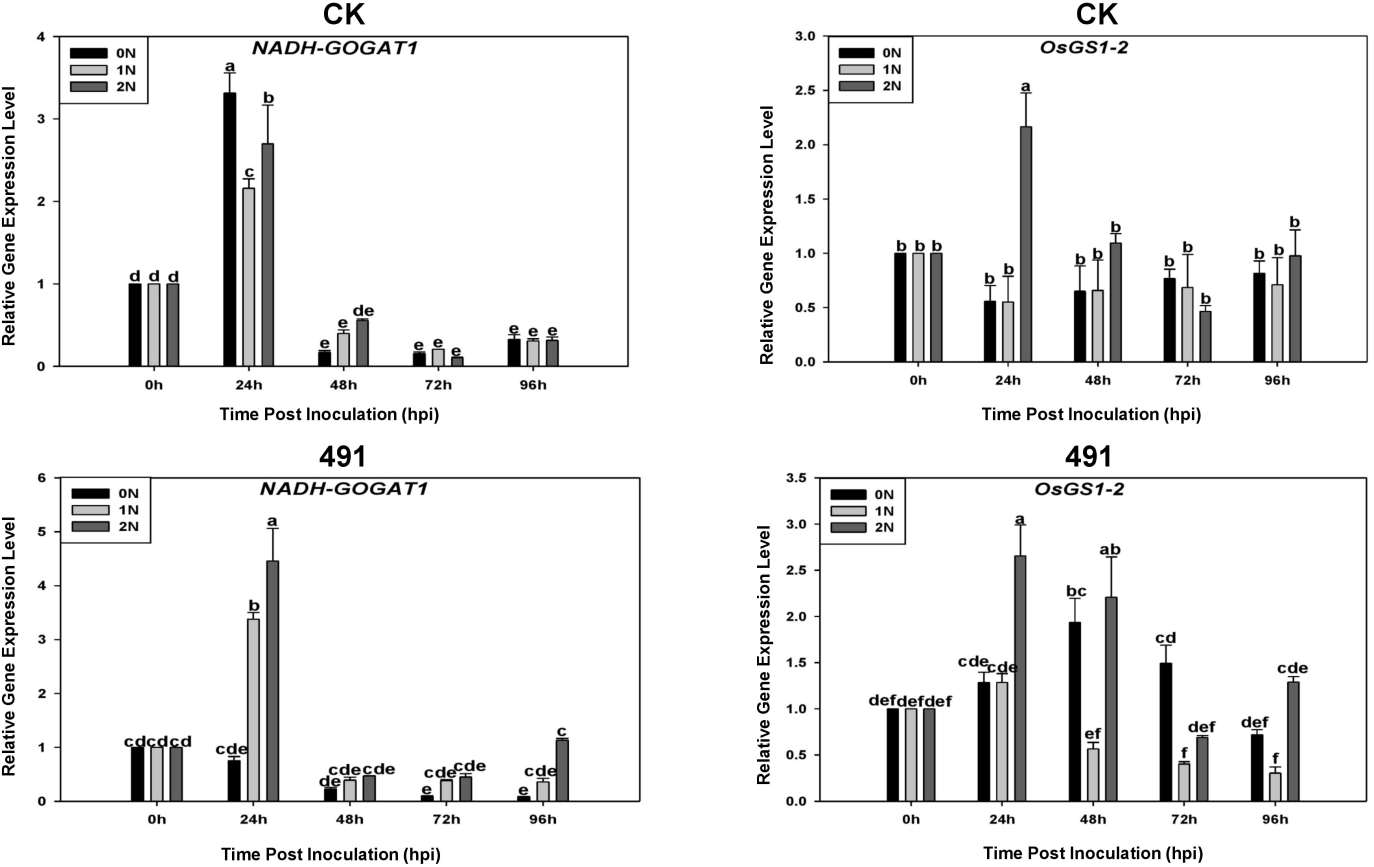
Expression patterns of NADH-GOGAT1 (left) and *OsGS1-2* (right) of the 4 varieties under 3 nitrogen conditions (0N, 1N and 2N) after 0h, 24h, 48h, 72h and 96h post inoculation of rice blast 95234I-1b. Error bars represent SD ± of the n = 5 biological replicates, and different letters above the bars showed statistical differences (P<0.05).

We also investigated the expression of key nitrogen assimilation genes. In the susceptible variety L-82, which served as our representative for gene expression analysis, OsGS1–2 expression was significantly and sustainably upregulated from 24 to 96 hpi under high nitrogen (2N), with a more modest increase under 1N (**Figure 7**). This sustained upregulation correlates with its higher susceptibility under 2N (NISI-2 > NISI-1) and aligns with previous reports linking increased glutamine synthesis to enhanced susceptibility under high fertilization.

## 4 Discussion

The use of high nitrogen (N) fertilizers enhances plant growth, resulting in more tillers and greater canopy density, which in turn favors disease spread. This creates a major issue in NIS studies, because it becomes difficult to separate the direct effects of nitrogen on disease resistance from the indirect effects of increased plant growth and altered morphology. Ballini et al. (2013) highlighted this confounding problem and developed an experimental system to minimize nitrogen’s impact on growth, thereby enabling investigation of the specific mechanisms underlying NIS. In the present study, the exact experimental conditions of Ballini et al. (2013) could not be replicated; therefore, modified nitrogen regimes were designed. Seedlings were grown in soil with lower baseline nitrogen than previous studies (e.g., Ballini et al., 2013, who used 40 mg L^−1^ total N). The nitrogen supplied during growth (1.1 × 10^−6^ g N seedling^−1^ day^−1^) was comparable to Ballini et al.’s low-N treatment (0.6 × 10^−6^ g N seedling^−1^ day^−1^) but substantially lower than in earlier studies (Yang et al., 2011). On the day prior to inoculation, seedlings received one of three treatments: 0N (control), 1N (100 mg L^−1^ NH_4_NO_3_), or 2N (200 mg L^−1^ NH_4_NO_3_). Under these conditions, plants showed no significant stunting, and baseline expression of nitrogen assimilation genes (NADH-GOGAT2, OsGS1-2) was similar across treatments in non-inoculated plants, indicating that our nitrogen regimes primarily affected defense responses rather than growth or constitutive nitrogen metabolism. A single nitrogen application 24 hours prior to inoculation represents a short-term pulse rather than a steady-state nutritional regime. This design was chosen to isolate the direct effects of nitrogen availability on early defense responses (peroxidase activity, lignin biosynthesis) during the critical pre-invasion and early infection stages, minimizing confounding effects from nitrogen-induced changes in plant growth, architecture, or tissue age. This contrasts with previous studies, such as Ballini et al. (2013) and Huang et al. (2017), which used sustained nitrogen treatments throughout plant development. While our approach provides mechanistic insights into acute nitrogen effects on immunity, it does not fully recapitulate field conditions where plants experience sustained nitrogen nutrition throughout growth. The temporal dynamics observed here should therefore be validated under long-term nitrogen regimes to assess their agricultural relevance.

Beyond nitrogen treatments, quantifying NIS remains a methodological challenge. Several researchers have developed or used stress-tolerance indices to determine relative drought tolerance, salinity tolerance, and other abiotic stress responses in order to examine genotype × environment interactions and yield stability (Tahmasebi et al., 2017; Morton et al., 2019). However, an index specifically designed to quantify resistance robustness under varying nitrogen conditions was proposed only recently (Frontini et al., 2021). In the present study, due to the unavailability of the LeAFtool software, the parameter LSM (lesion number per cm^2^ leaf area) was replaced by the traditional disease index (DI), which accounts for both lesion size and the number of infected leaves. The resulting NIS index (NISI) thus measures each variety’s resistance or susceptibility relative to the nitrogen effect on the whole population, and therefore differs from conventional robustness indices that do not account for nitrogen-dependent interactions.

In earlier studies, assessment of NIS (Ballini et al., 2013; Huang et al., 2017) was based mainly on lesion numbers. A complete disease evaluation, however, should include not only the number of lesions (successful penetration) but also lesion size (subsequent spread) and lesion type (successful infection) (Otani, 1952; Matsuyama and Dimond, 1973). Future work should aim to develop an integrated index encompassing all these components to better characterize nitrogen–pathogen–host interactions. To investigate NIS polymorphism, a genetically diverse rice panel was essential. The Rice Diversity Panel 2 (RDP2), originally developed for GWAS, was found to be highly diverse. Nine genetic subgroups and six geographic origins were represented by the 268 varieties analyzed. A wide range of NIS responses was observed, including positive NIS (increased susceptibility under high N), negative NIS (enhanced resistance under high N), and switching responses between 1N and 2N conditions. Two varietal groups are of particular breeding interest: (1) those maintaining robust resistance under elevated N, and (2) those exhibiting negative NIS. Using the RDP2 panel and HDRA genotyping data, several significant SNPs were mapped, including loci encoding F-box proteins and leucine-rich repeat receptor-like kinases. Such multidimensional NIS responses likely result from complex host–pathogen–nitrogen interactions, which are more intricate than typical biotic or abiotic stress models (Abro et al., 2013; Frontini et al., 2021). This complexity also helps to explain why NISI-1 and NISI-2 loci differ under distinct nitrogen regimes. The primary GWAS was performed on a panel from which constitutively resistant varieties were removed to enrich for NIS-responsive genotypes. While this filtering strategy was necessary to avoid masking of NIS-associated loci by major R genes, it may introduce biases such as altered allele frequencies. To address this concern, the full panel of 268 accessions was re-analyzed including baseline resistance (DI-0N) as a covariate. The major loci, including NIS4 and the chromosome 2 LRR-RLK cluster, remained significant (**Supplementary Table 2b**), confirming their robustness. However, the filtered panel likely underestimates the contribution of major R genes to NIS outcomes, and future studies should explore the interaction between NIS loci and R genes in near-isogenic backgrounds. While these candidate loci represent promising targets for future investigation, functional validation through mutant analysis, transgenic complementation, or near-isogenic line studies will be required to establish causality. This critical early time window aligns with the potential function of F-box proteins encoded at the *NIS4* locus, which are known to mediate rapid signaling events through targeted protein degradation. Varieties with favorable *NIS4* alleles may therefore be equipped to mount this early defense response upon pathogen detection, effectively utilizing available nitrogen for immediate cell wall fortification. The persistence of these associations in the full-panel analysis with DI-0N as a covariate (**Supplementary Table 2b**) indicates that they are not artifacts of genotype filtering and specifically capture nitrogen-responsive variation independent of constitutive resistance.

With increasing nitrogen supply, peroxidase (POD) activity and lignin-mediated resistance were enhanced in lines L-282 and L-29, reducing disease severity and resulting in negative NIS (**Figures 5**, **6**). In contrast, line L-157 exhibited reduced POD activity and lignin accumulation, leading to more severe symptoms and positive NIS. The response of L-82 was intermediate, showing transient POD induction at 1N but not sustained at 2N (**Figure 5**). The results of this study are not fully consistent with previous work showing that high nitrogen increases plant resistance but does not counterbalance the increased aggressiveness of pathogens, ultimately leading to higher susceptibility (Huang et al., 2017). Time-course analysis indicated that the first day after pathogen inoculation (1 dpi) was a crucial period. Varieties with negative NIS rapidly activated POD upon infection, effectively utilizing additional nitrogen for early defense, whereas varieties with positive NIS showed delayed responses. This is consistent with reports that nitrogen supplementation does not immediately induce major transcriptomic shifts in rice (Huang et al., 2017). Larger variations in POD activity at 2–3 dpi (48–72 h) further identified these as key windows for nitrogen-mediated defense.

High nitrogen also triggered NADH-GOGAT1 expression in infected L-82 leaves at 1 dpi, but induction subsequently declined, indicating that *Magnaporthe oryzae* may affect host nitrogen metabolism, as previously reported (Parker et al., 2009; Fernandez and Wilson, 2012; Huang et al., 2017). Ballini et al. (2013) and Le Gouis et al. (2000) found that nitrogen use efficiency (NUE) is associated with NIS, with high-NUE varieties exhibiting higher NIS. Furthermore, the NIS1 locus is syntenic to a meta-QTL controlling NUE in several monocots (Quraishi et al., 2011). Considering our observations, previous hypotheses, and temporal expression data, we propose that NIS outcomes are determined by two key stages: (1) Pre-invasion stage – varieties that can rapidly mobilize nitrogen toward defense upon pathogen detection exhibit little or negative NIS; and (2) Post-invasion stage – varieties that fail to redirect nitrogen promptly allow the pathogen to exploit elevated nitrogen, increasing susceptibility. Hence, both pre- and post-invasion processes determine whether nitrogen enhances resistance or susceptibility (Bolton, 2009; Seifi et al., 2013). After invasion, the fungus is likely to modify host nitrogen-assimilation pathways (e.g. OsGS1-2, NADH-GOGAT1, glutamine levels; Huang et al., 2017) and possibly the plant TCA cycle and GABA shunt (e.g. OsGAD3, OsGDH2; Takahashi et al., 2008). If varieties succeed in counteracting this increased pathogenicity, they will display little or negative NIS. Therefore, both pre-invasion and post-invasion processes are determinants for NIS outcomes.

The physiological and gene expression analyses were conducted on a limited number of varieties (four for POD/lignin assays, one for gene expression profiling). While these varieties were carefully selected to represent the spectrum of NIS phenotypes observed in our panel (**Supplementary Table 5**), the small sample size constrains the generalizability of the temporal dynamics reported here. The patterns observed, particularly the early POD activation in negative-NIS lines, should be validated in larger, independently selected sets of positive-NIS and negative-NIS varieties in future studies. Additionally, the gene expression analysis focused on a single susceptible variety (L-82); expanding this to include negative-NIS and resistant lines would provide a more complete picture of nitrogen-metabolism-defense crosstalk. Moreover, future work should include direct quantification of fungal biomass and ROS levels to further establish the causal relationship between nitrogen-mediated defense responses and resistance outcomes.

The two-stage model proposed in the Introduction is supported by our temporal data: negative-NIS lines rapidly activated POD and lignin deposition upon pathogen detection, consistent with effective pre-invasion defense mobilization. Conversely, positive-NIS lines exhibited delayed defense, allowing the pathogen to exploit elevated nitrogen post-invasion, as reflected by sustained OsGS1-2 expression. While these correlations are consistent with the model, direct experimental testing (e.g., metabolic flux analysis, genetic perturbation of NIS4 F-box genes) is required to establish causality.

## 5 Conclusion

**Figure 8:**
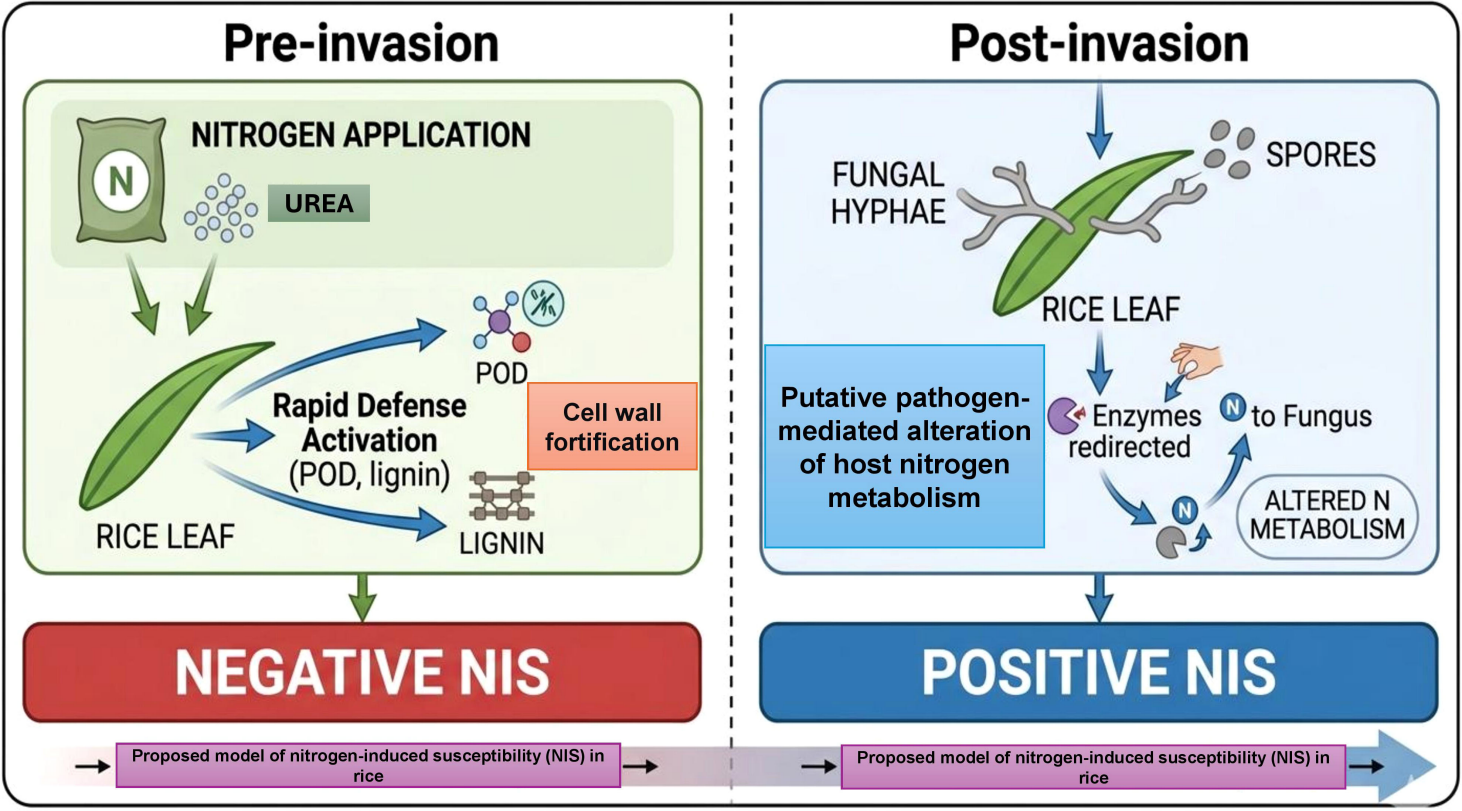
Proposed two-stage model of NIS. In the pre-invasion stage (left), nitrogen application potentiates rapid defense responses (peroxidase activity and lignin deposition) in resistant genotypes, leading to negative NIS (enhanced resistance). In the post-invasion stage (right), the pathogen manipulates host nitrogen metabolism; varieties that fail to counteract this manipulation become more susceptible (positive NIS). This model integrates the genetic, physiological, and temporal dynamics observed in this study.

To understand the genetic and physiological basis of NIS in rice, we analyzed multiple NIS-associated QTLs using a diverse rice population and a genome-wide association study (GWAS). We identified several loci/genes critical for defining NIS, including a new locus, NIS4, which contains two F-box protein genes, which may involve in the resistant against *M. oryzae*. This work expands and clarifies the genetic basis of this trait. The observed NIS polymorphism indicated that this common trait is not restricted to specific major subpopulations or regions and is therefore universally applicable to breeding programs. Physiological and molecular analyses further revealed that nitrogen responsiveness in a given variety is a time-sensitive, two-step process. The two-stage working hypothesis presented in the Introduction provides a conceptual framework for understanding how nitrogen availability interacts with defense timing to determine disease outcomes (**Figure 8**). Pre-invasion resistant cultivars rapidly redirect nitrogen to the cell wall through increased peroxidase activity and lignification, thereby preventing infection and resulting in negative NIS. During post-invasion, the pathogen’s impact on host nitrogen metabolism generates a metabolic “tug of war,” in which the outcome namely, the extent of disease is determined by the host plant’s ability to restrict pathogen nitrogen metabolism. In summary, the present study suggests that NIS can be conceptualized as a plastic phenotype influenced by host genetic susceptibility, pathogen strategy and nitrogen status, providing novel genetic resources and a physiological framework for developing high-yielding rice cultivars with broad-spectrum and nitrogen-resilient resistance against *M. oryzae* in the future. The genetic markers associated with *NIS4* and the major LRR-RLK cluster on chromosome 2 represent valuable targets for marker-assisted selection to stabilize blast resistance under high nitrogen fertilization regimes.

## Data Availability

The datasets presented in this study can be found in online repositories. The names of the repository/repositories and accession number(s) can be found in the article/**Supplementary Material**.
