# Supplementary material for: Genetics of polymorphism in nitrogen-induced-susceptibility of rice to Magnaporthe oryzae
Source: Front Plant Sci. 2026 May 7;17:1810580. doi: 10.3389/fpls.2026.1810580 (PMC13190176; doi:10.3389/fpls.2026.1810580)
Supplement: Supplementary file 1 [file DataSheet1.zip › Supplementary data sheet/Supplementary Table 3.DOCX]

Supplementary Table 3 Ranges of nitrogen induced susceptibility index (NISI) of varieties in our panel

| **NISI** | **＜-3** | **-3 ~ -1** | **-1 ~ 1** | **1 ~ 3** | **＞3** | **total** |
| --- | --- | --- | --- | --- | --- | --- |
| NISI-1 | 18 | 26 | 58 | 51 | 40 | 193 |
| NISI-2 | 4 | 34 | 65 | 39 | 51 | 193 |

**Note:** 193 varieties from ‘Rice Diversity Panel (RDP2)’ were screened for NIS investigation. NISI was the index of ‘nitrogen induced susceptibility (NIS)’ of varieties after infection of rice blast 95234I-1b responding to different nitrogen regimes (see Methods). NISI-1 and NISI-2 represented the quantity of varieties within the given ranges of NISIs of 1N vs 0N, and 2N vs 0N, respectively.
