# Supplementary material for: Genetics of polymorphism in nitrogen-induced-susceptibility of rice to Magnaporthe oryzae
Source: Front Plant Sci. 2026 May 7;17:1810580. doi: 10.3389/fpls.2026.1810580 (PMC13190176; doi:10.3389/fpls.2026.1810580)
Supplement: Supplementary file 1 [file DataSheet1.zip › Supplementary data sheet/Supplementary Table 4.DOCX]

Supplementary Table 4 Key genomic loci associated with nitrogen-induced susceptibility (NISI-1 and NISI-2) identified by GWAS.

| **Trait** | **Chromosome** | **Locus / Lead SNP** | **Position (Mbp)** | **PVE (%) Range** | **Key Candidate Gene(s)** |
| --- | --- | --- | --- | --- | --- |
| NISI-1 | 8 | NIS4 (SNP-8.5612001) | 5.61 - 5.62 | 9.8 - 13.2 | F-box proteins (*Os08g0197000*, *Os08g0197100*) |
| NISI-1 | 10 | SNP-10.16845000 | 16.84 - 16.87 | 10.1 -11.5 | Near previously reported NIS3 locus |
| NISI-2 | 2 | SNP-2.3073421 | 2.94 - 3.14 | 15.2- 20.1 | Leucine-rich repeat receptor-like kinases (LRR-RLKs) |
| NISI-2 | 6 | SNP-6.1245000 | ~12.45 | 8.5 - 9.1 | H3K27 methyltransferase (*Os06g0275500*) |
