# Supplementary material for: Genetics of polymorphism in nitrogen-induced-susceptibility of rice to Magnaporthe oryzae
Source: Front Plant Sci. 2026 May 7;17:1810580. doi: 10.3389/fpls.2026.1810580 (PMC13190176; doi:10.3389/fpls.2026.1810580)
Supplement: Supplementary file 1 [file DataSheet1.zip › Supplementary data sheet/Supplementary Table 5.DOCX]

Supplementary Table 5 Nitrogen-Induced Susceptibility Index (NISI) and Disease Index (DI) of the four varieties selected for physiological and gene expression analysis.

| **Variety code** | **NISI-1** | **NISI-2** | **DI-0N** | **DI-1N** | **DI-2N** |
| --- | --- | --- | --- | --- | --- |
| L-157 | 10.1 | 7.3 | 28.47 | 50.67 | 50.83 |
| L-82 | 2.0 | 6.7 | 37.18 | 42.96 | 63.98 |
| L-29 | -4.2 | -1 | 40.48 | 27.56 | 36.07 |
| L-282 | - | - | 18.5 | 6.8 | 8.22 |

Note: DI-0N, DI-1N and DI-2N were disease indexes of varieties after infection of rice blast 95234I-1b responding to 0N, 1N and 2N regimes, respectively (see Methods). NISI-1 and NISI-2 are the Nitrogen-Induced Susceptibility indices for 1N vs. 0N and 2N vs. 0N, respectively (see Methods).
*L-282 was excluded from GWAS due to stable resistance across all nitrogen treatments (disease state '1' in ≥3 replicates) but was included as a resistant benchmark for physiological assays; therefore, NISI values are not applicable.
