# Supplementary material for: Genetics of polymorphism in nitrogen-induced-susceptibility of rice to Magnaporthe oryzae
Source: Front Plant Sci. 2026 May 7;17:1810580. doi: 10.3389/fpls.2026.1810580 (PMC13190176; doi:10.3389/fpls.2026.1810580)
Supplement: Supplementary file 1 [file DataSheet1.zip › Supplementary data sheet/Supplementary Table 6.DOCX]

Supplementary Table 6 Haplotype analysis of the NIS4 locus (chromosome 8: 5.61–5.62 Mb).

| **Haplotype** | **Number of varieties** | **Mean NISI‑1 (± SD)** | **NISI‑1 range** |
| --- | --- | --- | --- |
| A | 67 | 5.2 ± 1.8 | 2.1 – 9.8 |
| B | 89 | 0.8 ± 0.6 | –1.5 – 2.5 |
| C | 37 | –2.1 ± 0.9 | –4.2 – –0.5 |

**ANOVA:** F = 12.4, df = 2, 190, P < 0.001
**Pairwise comparisons (Tukey HSD):** A vs. B: P < 0.001; A vs. C: P < 0.001; B vs. C: P < 0.01
