# Supplementary material for: Genetics of polymorphism in nitrogen-induced-susceptibility of rice to Magnaporthe oryzae
Source: Front Plant Sci. 2026 May 7;17:1810580. doi: 10.3389/fpls.2026.1810580 (PMC13190176; doi:10.3389/fpls.2026.1810580)
Supplement: Supplementary file 1 [file DataSheet1.zip › Supplementary data sheet/Supplementary Table 7.DOCX]

| **Trait** | **Metric** | **Correlation (r)** | **P‑value** |
| --- | --- | --- | --- |
| NISI-1 | ΔDI (1N‑0N) | 0.94 | <0.001 |
| NISI-1 | Reaction norm slope | 0.89 | <0.001 |
| NISI-1 | Log response ratio (1N/0N) | 0.91 | <0.001 |
| NISI-2 | ΔDI (2N‑0N) | 0.93 | <0.001 |
| NISI-2 | Reaction norm slope | 0.90 | <0.001 |
| NISI-2 | Log response ratio (2N/0N) | 0.92 | <0.001 |

Supplementary Table 7 Correlation of NISI with alternative metrics of nitrogen responsiveness. ΔDI: difference in Disease Index between the indicated nitrogen treatment and the 0N control. Reaction norm slope: linear regression coefficient of Disease Index against nitrogen level (0, 1, 2). Log response ratio: natural logarithm of (DI_N / DI_0N). All correlations were significant at P < 0.001 (Pearson’s correlation, n = 193 varieties).
